# Weed suppression and antioxidant activity of *Astragalus sinicus* L. decomposition leachates

**DOI:** 10.3389/fpls.2022.1013443

**Published:** 2022-11-16

**Authors:** Silin Liu, Wenhui Wang, Jiaoyun Chen, Zhiyu Ma, Youping Xiao, Zhongwen Chen, Ying Zhang, Xiao Du, Yinghui Mu

**Affiliations:** ^1^ College of Agriculture, South China Agricultural University, Guangzhou, China; ^2^ Zhongkai University of Agriculture and Engineering, Guangzhou, China; ^3^ Scientific Observing and Experimental Station of Crop Cultivation in South China, College of Agronomy/Ministry of Agriculture and Rural Affairs, Guangzhou, China

**Keywords:** milk vetch, goosegrass suppression, chemical compounds, phytotoxicity, antioxidant activity, acid compounds

## Abstract

*Astragalus sinicus* L. (milk vetch), a versatile plant that has a soil-enriching effect as green manure, is widely planted in the temperate zone of China. In previous experiments, milk vetch incorporated into the soil as green manure showed potential for goosegrass control. However, “what exactly happens at the chemical level?” and “what are the compounds that are potentially responsible for the phytotoxic effects observed during those previous assays?” In a recent study, *in vitro* phytotoxicity bioassays and chemical analyses of milk vetch decomposition leachates were carried out to explore the relationship between the temporal phytotoxic effects and the dynamics of chemical composition. For that, milk vetch decomposition leachates with a decay time of 12 h, 9 days, 12 days, 15 days, and 18 days were analyzed for organic compounds by liquid chromatography. The main results were as follows: (1) three compounds with goosegrass suppression potential produced during the decomposed process, i.e., 4-ethylphenol, N-acrylimorpholine, and allyl isothiocyanate. 2-Hydroxyethyl acrylate was present in the 12-h decomposition leachates but was at its highest concentration of 127.1 µg ml^−1^ at 15 days. (2) The cultures were configured according to the four concentrations of goosegrass-resistant active substances measured in the 15-day decomposition leachate and, as with the 15-day decomposition leachate, the mixture cultures inhibited 100% of goosegrass germination at the high concentrations (≥ 30%), which suggests that these substances have goosegrass suppression potential. (3) The high total phenolic content (302.8–532.3 mg L^−1^), the total flavonoid content (8.4–72.1 mg L^−1^), and the reducing activity of the decomposition leachates for different decay times may explain why the incorporation of milk vetch into the soil did not lead to peroxidation of goosegrass in the previous study. (4) Finally, the changes in acid fraction and total content (1.9–4.2 mg ml^−1^) for different decay times explain the variations in pH of the decomposition leachates, which, when discussed in conjunction with previous studies, may lead to changes in soil nutrient effectiveness and consequently affect crop growth. This study can provide a reference for green weed control research.

## 1 Introduction

Milk vetch is a biennial herb of the leguminous family *Astragalus* with a high yield and has been widely grown in southern China for centuries (Chang et al., 2022). Milk vetch is one of the most important green manures for increasing crop yields and preventing soil degradation (Chen et al., 2020; Zhou et al., 2020). The use of returned green manure to control weed growth in the field is feasible in many studies (Puig et al., 2013; Álvarez-Iglesias et al., 2018). Previous research has revealed that milk vetch turnover into the field as green manure can reduce the density of weed seed banks and increase the diversity and evenness of weed, which indicated the possibility of milk vetch applying for weed control (Tang et al., 2016). In paddy field trials, researchers found that the incorporation of milk vetch can suppress the germination of summer weeds (Utami et al., 2020). Previous works reported the feasibility of the use of milk vetch straw as phytotoxic green manure for weed control (Liu et al., 2022a). However, the foregoing studies were limited to epiphenomena or physiological phenomena and did not delve into the substances responsible for the goosegrass suppression potential of milk vetch.

The overuse of synthetic herbicides for weed control has led to many problems such as increased weed resistance, damage to soil microhabitats, and environmental pollution (Dhima et al., 2008; Bhadoria, 2011). In addition, goosegrass (*Eleusine indica* L.) has been reported to have developed resistance to several non-selective herbicides (Chuah et al., 2010; An et al., 2017). There is an urgent need to find other forms of weed control that can reduce the use of synthetic herbicides. Weed control can be achieved through the application of green manure. Green manures are highly competitive during the growing season and can be used to control weeds by releasing phytotoxic substances through the decay of residues back in the field (Liu et al., 2022b). Research on the application of green manure for weed control should include two components: the first being the physiological response of the plant and the second being the identification of phytotoxic substances and their mechanisms of action; linking the phenotypes to the phytotoxic compounds is a complete line of research for related studies. Taking a similar study as an example, researchers incorporated *Eucalyptus globulus* leaves into the field as green manures and found that it has potential as a material for bioherbicide (Puig et al., 2013; Puig et al., 2018a). The subsequent compound identification research indicated that phenolic and volatile organic substances were the ones released by *Eucalyptus globulus* leaves with weed suppression activity (Puig et al., 2018b). *Astragalus* spp. contain a variety of phytochemicals of medicinal value, including a high content of phenolics (Salehi et al., 2020), which may be involved in the role of antioxidant active substances in allelopathy effects. In addition, previous studies have shown that milk vetch does not inhibit the growth of goosegrass by causing peroxidation of cells, and we found that milk vetch decomposition leachates tend to polymerize and precipitate when exposed to light. By this phenomenon, we suspected that it might contain some amount of phenolics. In a study using Fourier transform infrared spectroscopy, researchers found that the water extract of the leguminous green manure milk vetch contained the highest levels of water-soluble organic matter, including higher concentrations of phenolics and more carboxylic acids than other green manures (Chang et al., 2020). The substances released from milk vetch that have an inhibitory effect on goosegrass growth remain unclear, and the question of whether milk vetch decomposition leachate has antioxidant activity is awaiting resolution.

To answer these questions, the objectives of this work were set out: (1) to identify goosegrass-resistant active substances in milk vetch decomposition leachate and clarification of the consistency between phytotoxic effects and the dynamics of allelochemicals released from milk vetch; (2) to study the goosegrass-resistant activity of four identified compounds; (3) to determine the total phenolic content and the total flavonoid content of the decomposition leachate and investigation of its antioxidant activity; (4) to measure the content and variation of common acid components in the decomposition leachate and explain the reasons for the dramatic change in pH value during decomposition. This study belongs to the preliminary research, mainly explaining the phenomenon of *in vitro* experiments and previous studies from the level of chemical compounds dynamic, which we assume can affect the growth of the plants.

## 2 Materials and methods

### 2.1 Plant material

Milk vetch (*Astragalus sinicus* L.) seeds for the experiment were purchased from Royal Garden Greening Engineering Co., Ltd. On 8 October 2021, we sowed 5 g of milk vetch seeds in the substrate [Jiffy^®^, consisting of 3 parts of peat soil and coconut husk, 1 part of vermiculite, and 1 of part perlite (v:v)] with a volume of 60 × 35 × 8 cm and placed them in an artificial climate incubator (temperature, 23 ± 2°C; humidity, 75%; 12-h/12-h light-dark cycle), keeping the substrate soil moist. To keep in line with the growing habits of the field, no fertilizer treatment was applied during the growth of milk vetch seedlings. The seedlings were planted in Jiffy substrate before harvest. On 8 December of the same year, before the milk vetch flowered, the aboveground parts of milk vetch were harvested with fresh and dry yields of 1.01 and 0.32 Kg m^-2^, respectively. The harvested milk vetch was vacuum freeze-drying at −50°C and 100 Pa for 48 h and crushed; the powder was sealed and stored in a −20°C refrigerator for later use. Goosegrass seeds were collected in the Experimental Base of South China Agricultural University (23°14′18.42″ N, 113°38′8.06″ E).

### 2.2 Experiment and treatment design

We used milk vetch straw powder as material, mixed it according to the following ratio: powder, fresh paddy soil, and ultra-pure water (1:1:30 by weight), and put them into a plastic bottle with an inner lid. Soil solution without powder was used as the control (CK), and each treatment set three duplications. The bottle was placed in a constant temperature shaker at 28°C for 200 rpm for 12 h, 9 days, 12 days, 15 days, and 18 days to obtain the decomposition leachates. All the decomposition leachates were stored in a −40°C refrigerator and were used for the test together after the last decomposition.

#### 2.2.1 Identification of goosegrass-suppressed composition in milk vetch by LC

4-Ethylphenol, 2-hydroxyethyl acrylate, and N-acrylimorpholine were obtained from MACKLIN^®^ (Shanghai, China), with the purity of 97%, 97%, and 98%, respectively. Allyl isothiocyanate was obtained from ChromaDex^®^ (J&K Scientific, China), with a purity of 99%. The above compounds at 0.01 g were accurately weighed, and the standard solutions at 0.1 mg ml^−1^ were prepared using chromatographically pure methanol and were then filtered by 0.22-µm microporous membrane. All the decomposition leachates were diluted twice for better separation and were filtered through filter paper and further filtered through the 0.22-µm microporous membrane and then analyzed by LC-SPD-20A according to Su et al. (2022a). LC-20A (Shimadzu, Japan) with a Prominence SPD-20A UV-VIS detector was used for the detection. 4-Ethylphenol and allyl isothiocyanate were detected under the same conditions, with an Agilent TC-C18 (250 mm, 4.6 mm, and 5 µm) chromatographic column, with chromatographic conditions as follows: mobile phase A: methanol (chromatographic purity), mobile phase B: ultrapure water, A:B = 50%:50%; elution method: equal gradient elution; detection wavelength was 235 nm; column temperature: 30°C; injection volume: 20 µl; flow rate: 0.7 ml min^−1^. The detection conditions for N-acrylimorpholine were similar to 4-ethylphenol and allyl isothiocyanate, but an additional column was coupled in series for better separation, and the flow rate was downgraded to 0.45 ml min^−1^ accordingly. The detection for 2-hydroxyethyl acrylate were adopting an Agilent TC-C18 (250 mm, 4.6 mm, and 5 µm) chromatographic column, with chromatographic conditions as follows: mobile phase A: methanol (chromatographic purity), mobile phase B: ultrapure water, A:B = 10%:90%; elution method: equal gradient elution; detection wavelength was 210 nm; column temperature: 30°C; injection volume: 20 µl; flow rate: 0.9 ml min^−1^. Data were processed on Origin 2018 software (Originlab^®^). Compounds with goosegrass-suppressed potential were identified by comparing their elution order and UV-VIS spectra with authentic standards. Peak purity was checked by the software contrast facilities. Goosegrass-suppressed active compound quantification was achieved by the absorbance recorded in the chromatograms relative to external standards, using the following equation:


$$C\left(c\right)=\frac{A\left(c\right)}{A\left(Std\right)}\times C\left(Std\right)\times 2$$

where *C(c)* is the concentration of the compound contained in the decomposition leachates, *A(c)* is the peak area of the compound in the sample chromatogram, *A(std)* is the area of the peak for the standard in the reference chromatogram, *C(std)* is the concentration of the standard in the reference solution, and 2 is the dilution factor. This procedure was performed in triplicate for each sampling time.

#### 2.2.2 Phytotoxic potential of identified compounds in decomposition leachates

The pH and conductivity of the filtered decomposition leachates were determined directly using a pH meter (SX711, Shanghai) and conductivity meter (SW-301, Guangdong). The master batch of the culture solution was prepared according to the concentration of each compound in 15-day decomposition leachate and diluted in a gradient of 2%, 5%, 30%, and 80% as the culture solution for the *in vitro* goosegrass suppression activity assay respectively. Another master batch was prepared at the concentration of the four compounds in the 15-day decomposition leachates and similarly diluted into the above gradient culture for the *in vitro* goosegrass suppression activity trials, to observe the goosegrass suppression effect of the mixed compound culture. We used 2.2% sodium hypochlorite to disinfect the goosegrass seeds for 10 min, adopted the double-layer filter paper method, inoculated 30 seeds of goosegrass into Petri dishes, and treated them with the treatment solution. Sterile water treatment was used as a control (CK), and each treatment was set up in five replicates. The inoculated petri dishes were placed in an artificial climate incubator (KES^®^, Guangdong) at a temperature of 26 ± 2°C, relative humidity of 75%, and a light-dark cycle of 12 h/12 h. Water was replenished, and the germination number was recorded every day. The emergence of the radicle was taken as the indicator of germination. When the germination rate remained unchanged for 3 days, the recording was stopped.

#### 2.2.3 Total phenolic and flavonoid content and the antioxidant activity of milk vetch decomposition leachates

Total phenolic content and total flavonoid content were determined by using a colorimetric method described previously (Dewanto et al., 2002), total reduction capacity determination as referred by Shon et al. (2004), and 1,1-diphenyl-2-picrylhydrazyl (DPPH) radical scavenging capacity determination as referred by Merghem et al. (2020). ABTS^+^-free radical scavenging capacity assay as referred by Binsan et al. (2008). The total phenolic content, total flavonoid content, and the reducing activity of the decomposition leachates were measured as equivalent to gallic acid, rutin, and vitamin C, respectively.

#### 2.2.4 Changes in acid fraction and concentration of milk vetch decomposition leachates

Acetic acid, citric acid, ellagic acid, tartaric acid, and propionic acid were purchased from MACKLIN^®^ (Shanghai, China), with a purity of 99.7%, 99.5%, 99.5%, 95%, and 99.5%, respectively. Oxalic acid and succinic acid were purchased from Aladdin^®^ (Shanghai, China), with a purity of 99% and 96%, respectively. Fumaric acid was purchased from OKA^®^ (Beijing, China), with a purity of 95%. The above compounds at 0.01 g were accurately weighed, and the standard solutions at 0.01 mg ml^−1^ were prepared using H_3_PO_4_ (0.02 mol L^−1^; mobile phase) and were then filtered by 0.22-µm microporous membrane. All the decomposition leachates of milk vetch were filtered through filter paper and further filtered through the 0.22-µm microporous membrane and then analyzed by LC-SPD-20A with an HP-C18 (250 mm, 4.6 mm, and 5 µm) chromatographic column, with chromatographic conditions as follows: mobile phase was 100% H_3_PO_4_ (0.02 mol L^−1^); elution method: equal gradient elution; detection wavelength was 220 nm; column temperature was 37°C; injection volume was 10 µl; flow rate was 1.4 ml min^−1^. Data were processed on Origin 2018 (Originlab^®^). Acid fraction quantification was achieved by the absorbance recorded in the chromatograms relative to external standards using the following equation:


$$C\left(c\right)=\frac{A\left(c\right)}{A\left(Std\right)}\times C\left(Std\right)$$

### 2.3 Data collection

In the goosegrass-suppressed active substance identification assay, we determined three duplications of each time point decomposition leachates, and the average of the chromatographic signals from the three replicates at each time point was used to re-plot the signal intensity of the substance content. In the bioassay test, we determined six germination indicators, i.e., germination potential, germination rate, germination index, vital index, plant height, and fresh weight. Ten seedlings with uniform growth were selected from each treatment to measure plant height. Twenty plants were randomly selected from three of the five replicates to determine fresh plant weight (the number of samples was reduced by a certain proportion, and the final result was multiplied by the corresponding multiple after weighing when the sample was insufficient). Germination-related indicators were calculated according to the following formula:


$$Gp=\frac{number\;of\;germinatedseedsat2d}{numberof\;testedseeds}\times 100\%;$$

$$Gr=\frac{numberofnormalger\min ationseeds}{numberoftestedseeds}\times 100\%;$$

Vi=s×Gi,(s​means​plant​height​here);

$$Gi=\sum (\frac{Gt}{Dt}),(Gt:Germination\;numberatdifferentday,Dt:Thestatisticalnumberofdays);$$

where *Gr* is the germination rate, *Gp* is the germination potential, *Gi* is the germination index, and *Vi* is the vital index.

The allelopathic effects of milk vetch were evaluated using means of response index (RI). When T ≥ C, $RI=1-\frac{C}{T}$ ; T < C, $RI=\frac{T}{C}-1$ (C: the result of CK; T: the result of treatment, use the mean values as the result). RI > 0, indicating allelopathic promotion; RI < 0, indicating allelopathic inhibition; absolute value was consistent with the degree of allelopathy (Williamson and Richardson, 1988). RI accumulate value was the sum of six RI values of germination indicators. RI accumulate values of each indicator were calculated, and the allelopathic effects of milk vetch were evaluated comprehensively. RI accumulate values reach −6, indicating that germination and growth of goosegrass were completely inhibited.

### 2.4 Statistical analysis

Replicated experiments were conducted in a completely randomized design. For the parameters of goosegrass *in vitro* trials, determination of total phenolic content, and antioxidant tests, statistical significance was analyzed by one-way ANOVA. We verified normality and homogeneity of variance using the Shapiro–Wilk test and Levene’s test, respectively. Significant differences were further compared using the *post-hoc* Fisher LSD test. SPSS 24 was used for significance analysis of the data, which were expressed as mean ± standard deviation. GraphPad Prism 7 was used to plot. For parameters of pH and EC between CK and decomposition leachates, the statistical significance of treatment effects was determined by independent samples Student’s t-test (*P* ≤ 0.05)

## 3 Result and discussion

### 3.1 Appearance, pH, and electrical conductivity of different decomposition leachates

The pH value of the decomposition leachates decreased by 24.1%, 37.8%, 40.3%, 47.2%, and 43.2% at 12 h, 9 days, 12 days, 15 days, and 18 days, respectively, compared with that of the CK (**Table 1**). The EC of the 12-h decomposition leachate was 10.5% higher than that of the CK. In contrast, the EC of 9-day decomposition leachate was 23.7% lower than that of CK. The EC of 12- to 18-day decomposition leachates showed no differences compared with that of CK. The increase in EC at 12-h decomposition leachate was mainly due to the release of chemical elements from milk vetch, whereas the subsequent decrease may be due to the uptake of the elements by the colony and the chelation of certain elements such as manganese ions by organic substances such as oxalic acid and citric acid. The trend in EC changes was consistent with the findings of the previous study (Liu et al., 2022). Researchers found that in the paddy-upland rotation system, milk vetch incorporation as green manure decreased soil pH value, which is consistent with the findings of this study (Zhong et al., 2021). We estimated the lowest pH value at 15 days may be related to the increased acid content, whereas the increased pH value at 18 days may be related to the decrease in acid, and whether the pH can be restored to its pre-decomposition state needs to be further investigated.

**Table 1:** pH values and electrical conductivity of CK and decomposition leachates from different decay durations.

Decay duration	pH values			Electrical conductivity		
	CK	Decomposed liquids	Sig.	CK	Decomposed liquids	Sig.
12 h	7.9 ± 0.1	6.0 ± 0.2	***	3.8 ± 0.05	4.2 ± 0.1	*
9 days	7.4 ± 0.1	4.6 ± 0.2	***	3.8 ± 0.01	2.9 ± 0.5	*
12 days	7.2 ± 0.02	4.3 ± 0.3	***	3.7 ± 0.03	3.5 ± 0.2	ns
15 days	7.2 ± 0.08	3.8 ± 0.8	***	3.7 ± 0.07	3.8 ± 0.1	ns
18 days	7.4 ± 0.05	4.2 ± 0.3	***	3.7 ± 0.02	3.6 ± 0.1	ns

Values denote the means of three replicates. Asterisks denote significant effects between CK and milk vetch decomposition leachates: * P ≤ 0.05 and ***P ≤ 0.001; otherwise, ns (P > 0.05); independent samples t-test.

### 3.2 Dynamics of goosegrass-suppressed potential substances from different decomposition leachates

As shown in **Table 2** and **Figure S1**, 4-ethylphenol, N-acrylimorpholine, and allyl isothiocyanate appeared on the 9 days and in subsequent leachates. 2-Hydroxyethyl acrylate was present in the 12-h decomposition leachates, with a concentration of 1.0 µg ml^−1^. The highest concentrations of 4-ethylphenol and 2-hydroxyethyl acrylate at different decomposition times were at 15 days, with concentrations of 20.2 and 127.1 µg ml^−1^, respectively, whereas the highest concentrations for allyl isothiocyanate and N-acrylimorpholine were on 18 days, with concentrations of 32.4 and 247.2 µg ml^−1^, respectively. Goosegrass-suppressed active substances have a pattern of “Hormesis effect”, i.e., dose-dependent, which has been reported in related articles (Álvarez-Iglesias et al., 2018; Eladel et al., 2019; Ojija et al., 2019). The green manure straw takes time to release the goosegrass-resistant potential substances and these substances may be produced with concomitant degradation, which gives rise to a peak in concentration, preceded by a gradual increase in goosegrass suppression potential and followed by a decrease. In a study exploring the effects of *Brassica napus* decomposition on the growth of mung beans, the decomposition period changed the phytotoxic effect of residues; more inhibitory effects were shown at 14 days of decomposition, whereas decomposition for 21 days reduced the inhibitory effect (Mehmood et al., 2018). Puig et al. (2013) suggested adopting a relay planting of maize after 12 to 15 days from eucalyptus incorporation, as the early decomposition of eucalyptus leaves returned as green manure inhibits maize growth. The decomposition patterns, and the persistence of phytotoxic chemicals differ between plants. Researchers found a rule in the cover crop decomposition studies that the phenolic acids released from cereal rye increased in soils during the first 3–7 days after cereal rye termination and then decreased to initial concentrations after 56 days (Otte et al., 2020). Previous studies have shown that the return of milk vetch to the field promoted crop growth, so our focus should be on the point in time when the concentration of goosegrass-resistant active substances in the milk vetch decomposition leachate was at its highest.

**Table 2:** The concentration of goosegrass-suppressed active substances in different decay duration decomposition leachates.

Compound	Concentration (µg ml^−1^)				
	12 h	9 days	12 days	15 days	18 dayd
4-Ethylphenol	n.d. e	8.9 ± 0.2 d	14.2 ± 0.4 c	20.2 ± 0.09 a	16.2 ± 0.3 b
Allyl isothiocyanate	n.d. d	10.5 ± 1.7 c	26.2 ± 1.1 b	25.3 ± 2.3 b	32.4 ± 2.2 a
N-acrylimorpholine	n.d. e	111.4 ± 0.2 d	178.1 ± 1.6 c	230.1 ± 1.1 b	247.2 ± 0.9 a
2-Hydroxyethyl acrylate	1.0 ± 0.03 d	64.2 ± 4.6 c	89.9 ± 3.9 b	127.1 ± 2.7 a	94.5 ± 4.1 b

All data were subjected to analysis of variance with the post-hoc LSD test. Different letters denote significant differences among different treatments at P ≤ 0.05. Data represent mean values of three replicates ± SD.

“n.d.” indicates that the test and result are below the method detection limit. The detection limits (DL) of 4-ethylphenol, allyl isothiocyanate, and N-acrylimorpholine were 0.3, 0.5, and 0.3 µg ml^−1^ respectively. Statistical calculations were performed using one-half of the DL concentration.

### 3.3 Identified substances on the germination and growth of goosegrass

All four identified compounds, i.e., 4-ethylphenol, allyl isothiocyanate, N-acrylimorpholine, and 2-hydroxyethyl acrylate, inhibited the growth of goosegrass to varying degrees at high concentrations (≥30%), especially N-acrylimorpholine and 2-hydroxyethyl acrylate (**Pictures S1C, D**). The weaker inhibition of goosegrass by allyl isothiocyanate and 4-ethylphenol (**Pictures S1A, B**) may be related to their lower concentrations. The four mixtures inhibited the goosegrass germination rate of 14.4% and 41.3% at 2% and 5% decomposition leachate treatments, respectively, which was stronger than the same concentration of the single compound (**Picture S1E**).

**Figure 1:**
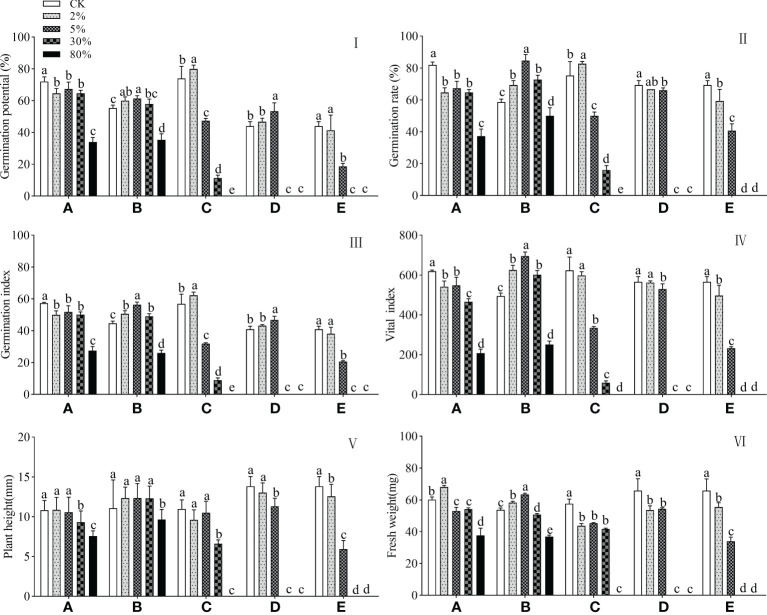
Effect of different identified substances on germination and growth of goosegrass. Different letters above each group of the bar chart denoted significantly different among different concentration treatments of identified substance at *P* < 0.05 by LSD test, respectively. Bars represent 10, 3, and 5 repetitions ± SD in plant height, fresh weight, and germination-related parameters, respectively. **(A)** 4-Ethylphenol. **(B)** Allyl isothiocyanate. **(C)** N-acrylimorpholine. **(D)** 2-Hydroxyethyl acrylate. **(E)** Mixture of **(A-D)**.

4-Ethylphenol is a strong odorant produced by microbial activity that is described as off flavors in several foods (Santamaría et al., 2018), especially wine production (Nieto-Rojo et al., 2014; Branco et al., 2021). Low concentrations (0.2 mM) of 4-ethylphenol promote crop growth and have the ability to disrupt the cell membranes of pathogens, so it can be used as an oomycete biological control agent for soybean root rot and tobacco black shank diseases (Ge et al., 2021). 4-Ethylphenol at concentrations ≥ 2% had a significant inhibitory effect on goosegrass germination (**Figures 1I–IV, A**), especially at high concentrations (≥ 30%), but the plant height was not affected at 2% concentration (**Figure 1V, A**). Treatment with 4-ethylphenol at 2% concentration increased the fresh weight of goosegrass seedlings by 13.3% compared with CK (**Figure 1VI, A**), which was inhibited with increasing concentrations, with 37.4% inhibition at 80% concentration.

Allyl isothiocyanate is a naturally occurring plant volatiles with insecticidal properties and can be 100% inhibition against larval emergence from spotted-wing drosophila eggs in blueberry fruits after 24 h of exposure under an air level of 0.5 µl L^−1^ (Jabeen et al., 2021). Using a novel analysis of protein target-assisted computational tools for homology modelling, the researchers found that the antibacterial potency of Brassica nigra essential oil is due to the preferable binding efciency of allyl isothiocyanate, the major active ingredient of B.nigra essential oil (Mandal et al., 2021). In this study, allyl isothiocyanate elevated goosegrass germination at concentrations ≤ 30%, with the strongest promotion of 44.3% at 5% treatment (**Figures 1I–IV, B**), whereas the germination index, fresh weight, and plant height (**Figures 1III,V,VI, B**) of goosegrass were inhibited by 41.6%, 25.7%, and 12.6%, respectively, at 80% concentration.

N-acrylimorpholine is a water-soluble compound with an unsaturated carbon-carbon double bond and is an important raw material for the synthesis of polymers. Because of its non-toxic nature and good biocompatibility, homopolymers of N-acrylomorpholine can be used as drug retardants, water treatment agents, and cosmetic support agents (Wang and Zhang, 2003). Dimethomorph, a new type of fungicide, for the control of diseases caused by fungi of the oomycetes of the *Subphylum Flagellaria* (Lu et al., 2019), has a similar structure to N-acrylimorpholine. The treatment with a 2% concentration N-acrylimorpholine elevated germination-related indicators, except for the vital index of goosegrass (**Figures 1I–III, C**). Briefly, the germination rate, germination potential, and germination index of goosegrass were improved by 9.7%, 8.1%, and 9.3%, respectively, and the vital index was inhibited by 4.1%. At 30% concentrations treatment, all germination indicators, plant height, and fresh weight of goosegrass were significantly (*P* < 0.05) inhibited. Briefly, the germination rate, germination potential, germination index, vital index, plant height, and plant weight of goosegrass were inhibited by 78.8%, 84.7%, 84.3%, 90.5%, 39.7%, and 27.7%, respectively. One hundred percent of the inhibition of goosegrass germination was recorded at 80% concentration of N-acrylimorpholine treatment (**Figures 1I–V, C**).

2-Hydroxyethyl acrylate is a raw material for the development of high-performance composite biomaterials in the materials field (Bahmani et al., 2019). No study is showing that 2-hydroxyethyl acrylate inhibits weed growth. In this study, 2-hydroxyethyl acrylate did not affect goosegrass germination at 2% concentration treatment (**Figures 1I–IV, D**). Compared with CK, treatment with a 5% concentration of 2-hydroxyethyl acrylate increased the germination potential and germination index of goosegrass by 21.2% and 14.3%, respectively (**Figures 1I,III, D**), but the germination rate and vital index were inhibited by 4.8% and 6.5%, respectively (**Figures 1II,IV, D**). 2-Hydroxyethyl acrylate inhibited goosegrass fresh weight, as reflected by 18.5% and 17.4% decreased goosegrass fresh weight at 2% and 5% concentration treatments, respectively (**Figure 1VI**). Similarly, the plant height of goosegrass decreased with increasing concentrations of 2-hydroxyethyl acrylate (**Figure 1V, D**), with 5.9% and 18.2% reduction in plant height for 2% and 5% concentrations, respectively.

The mixture of the four components inhibited the germination and growth of goosegrass, with all concentrations except for the 2% treatment, which did not inhibit the germination potential and germination index of goosegrass. The germination and growth of goosegrass were completely inhibited at concentrations of ≥ 30% of the four components (**Figures 1I–VI, E**).

**Figure 2:**
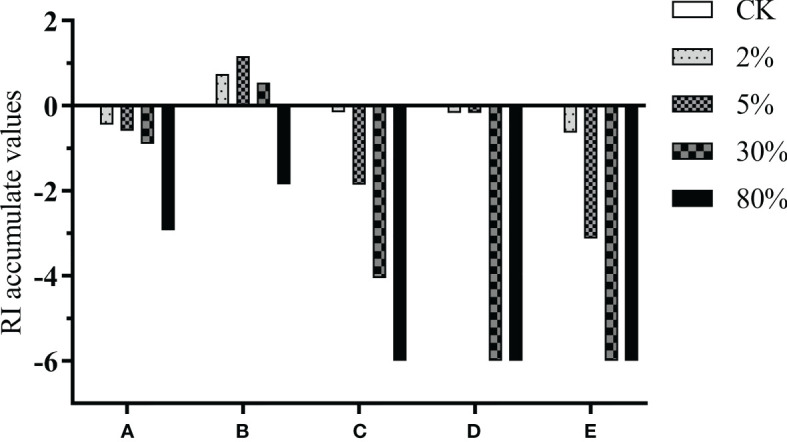
RI accumulation value of different identified substances on germination and growth of goosegrass. **(A)** 4-Ethylphenol. **(B)** Allyl isothiocyanate. **(C)** N-acrylimorpholine. **(D)** 2-hydroxyethyl acrylate. **(E)** Mixture of **(A)** to **(D)**.

The RI accumulation value plot shows that 4-ethylphenol (**Figure 2A**) inhibited the growth of goosegrass as the concentration increased. Concentrations of allyl isothiocyanate (**Figure 2B**) up to 30% improve germination and growth of goosegrass but inhibit it at 80%. The growth inhibition in goosegrass by N-acrylimorpholine (**Figure 2C**) increased with increasing concentration. The RI values of 2% and 5% 2-hydroxyethyl acrylate treatments were −0.17, whereas the RI values of 30% and 80% treatment reached −6, which suggested that the goosegrass suppression potential of 2-hydroxyethyl acrylate (**Figure 2D**) was much stronger at high concentrations than at low concentrations. The inhibitory effect of the mixture of the four components (**Figure 2E**) on the growth of goosegrass increased with increasing concentration.

### 3.4 Total phenol and flavonoid content and reducing activity of the decomposition leachates

#### 3.4.1 Total phenolic and total flavonoid content

**Figure 3:**
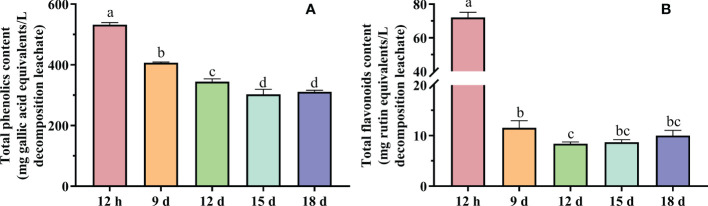
Total phenolic **(A)** and total flavonoid **(B)** content of different decay time decomposition leachates. Different letters denoted significantly different (*P* < 0.05) among different decomposition leachates of decay durations. Bars represent three repetitions ± SD.

The genus *Astragalus* is the largest in the *Fabaceae* family, which is known to contain different metabolites such as flavonoids, saponins, and polysaccharides (Bratkov et al., 2016). Fourteen-day-old milk vetch seedlings could contain up to 7.3 mg g^−1^ dry weight of total phenolics (Zhao et al., 2022). The 12-h decomposition leachate contained total phenolics of 532.3 mg L^−1^ (**Figure 3A**) and total flavonoid of 72.1 mg L^−1^ (**Figure 3B**), which is higher than other decomposition leachates. The total phenolic content of 15-day decomposition leachates and the total flavonoid content of 12-day decomposition leachates were the lowest, with a total phenolic content of 302.8 mg L^−1^ and a total flavonoid of 8.4 mg L^−1^. The total phenolic content on 18 days increased slightly but was similar to 15-day decomposition leachate, similarly the flavonoid content on 15 and 18 days increased compared with that on 12-day decomposition leachate. Changes in total phenolics and flavonoid content may be related to the degradation of extant phenolics and the resynthesis of other phenolics or flavonoids. The change in phenolic content was consistent with (Puig et al., 2018), i.e., treatments with short decay times contained the highest total phenolic content, which decreased as decay time increased.

#### 3.4.2 Total reducing capacity

**Figure 4:**
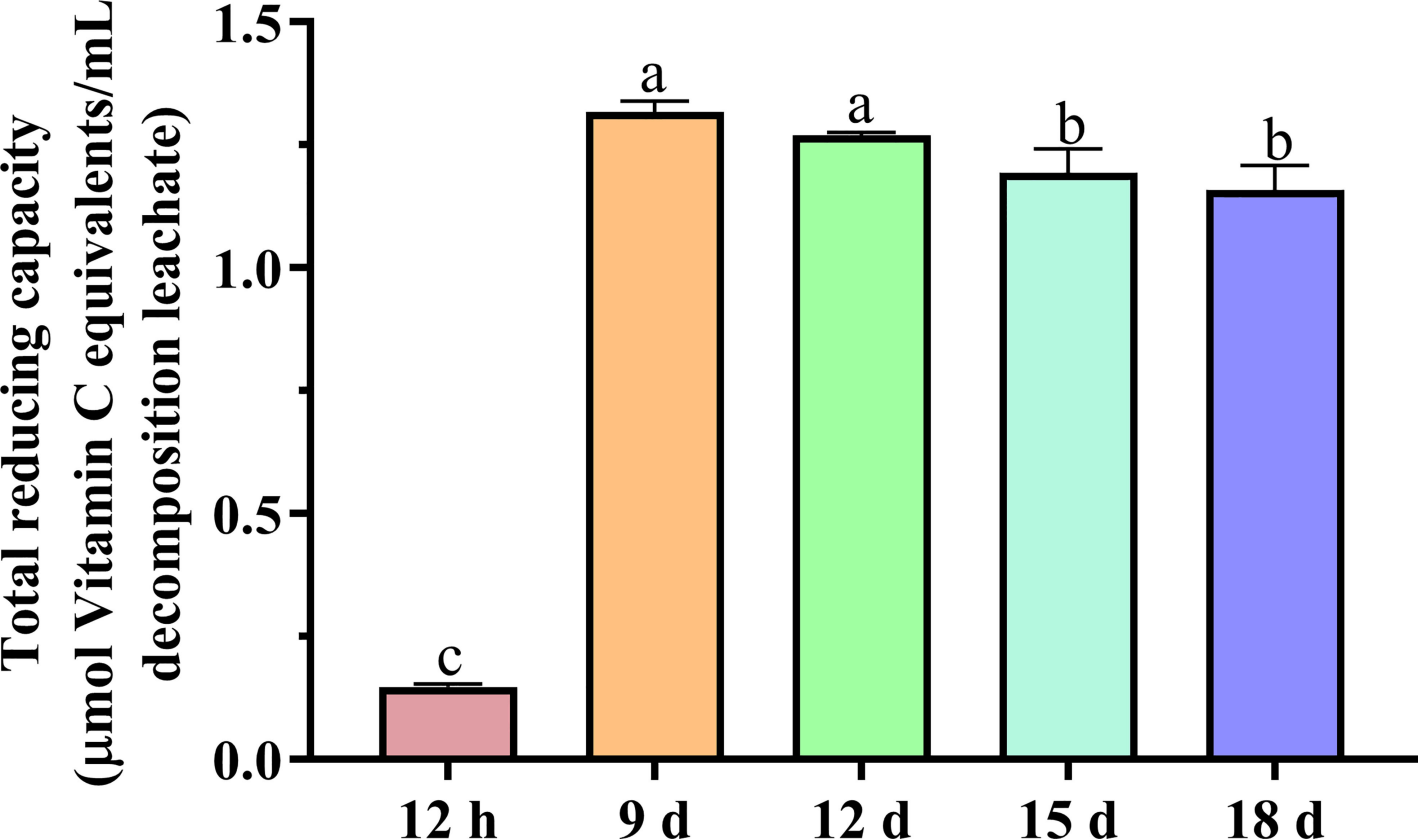
Total reducing capacity of the decomposition leachates at different decay times. Different letters denoted significantly different (*P* < 0.05) among different decomposition leachates of decay durations. Bars represent three repetitions ± SD.

The 12-h decomposition leachate had the lowest total reducing capacity (0.15 μmol of vitamin C/ml decomposition leachate). After 9, 12, 15, and 18 days of decomposition, the total reducing capacity had increased to 1.32, 1.27, 1.19, and 1.16 μmol of vitamin C/ml decomposition leachate, respectively (**Figure 4**). The increase in total reducing capacity was statistically significant between 12 h and the other decomposition leachates (LSD test, *n* = 3, *P* < 0.05). Using an *in vitro/in vivo* model of liver injury, three flavonoids with antioxidant activity were isolated from *Astragalus spruneri* Boiss. (Fabaceae), which reduced malondialdehyde (MDA) production by reducing lipid membrane peroxidation and restoring glutathione levels and the activity of catalase (CAT) and superoxide dismutase (SOD) (Kondeva-Burdina et al., 2018). Previous studies have shown that the appropriate amount of milk vetch can increase the activity of the protective enzyme system and reduce the MDA content of maize (Liu et al., 2022c), which may be related to the increased total reducing the capacity of milk vetch during decomposition.

#### 3.4.3 ABTS^+^ and DPPH-free radical scavenging capacity

**Figure 5:**
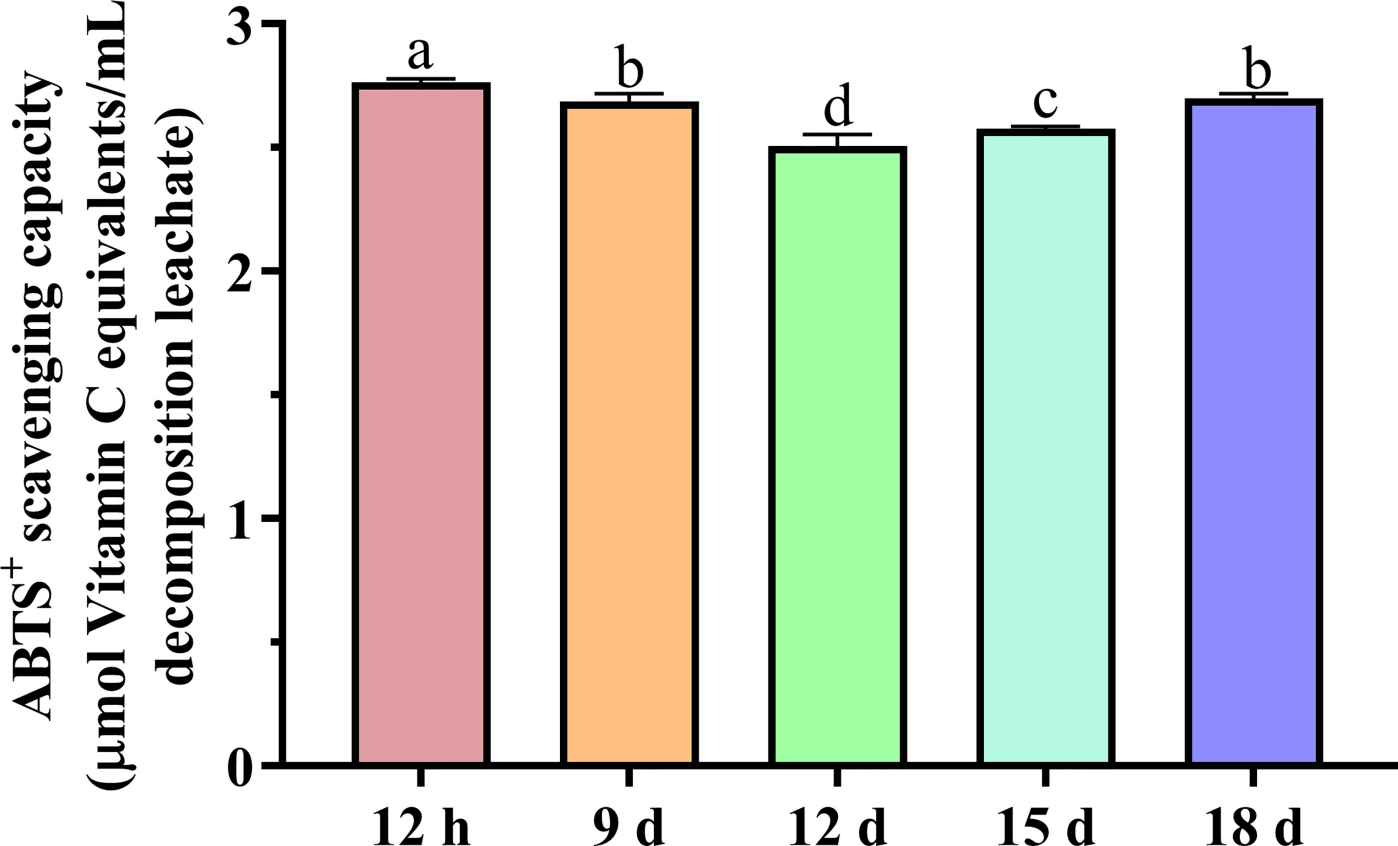
ABTS^+^ scavenging capacity of the decomposition leachates at different decay times. Different letters denoted significantly different (*P* < 0.05) among different decomposition leachates of decay durations. Bars represent three repetitions ± SD.

ABTS^+^ free radical scavenging capacity was close to vitamin C of 500 mg L^−1^ for all lengths of decay (**Figure 5**), with the 12-h decomposition leachate being the strongest (2.76 μmol of vitamin C/mL decomposition leachate), followed by the 18-, 9-, 15-, and 12-day decomposition leachates (2.70, 2.69, 2.58, and 2.51 μmol of vitamin C/mL decomposition leachate, respectively).

**Figure 6:**
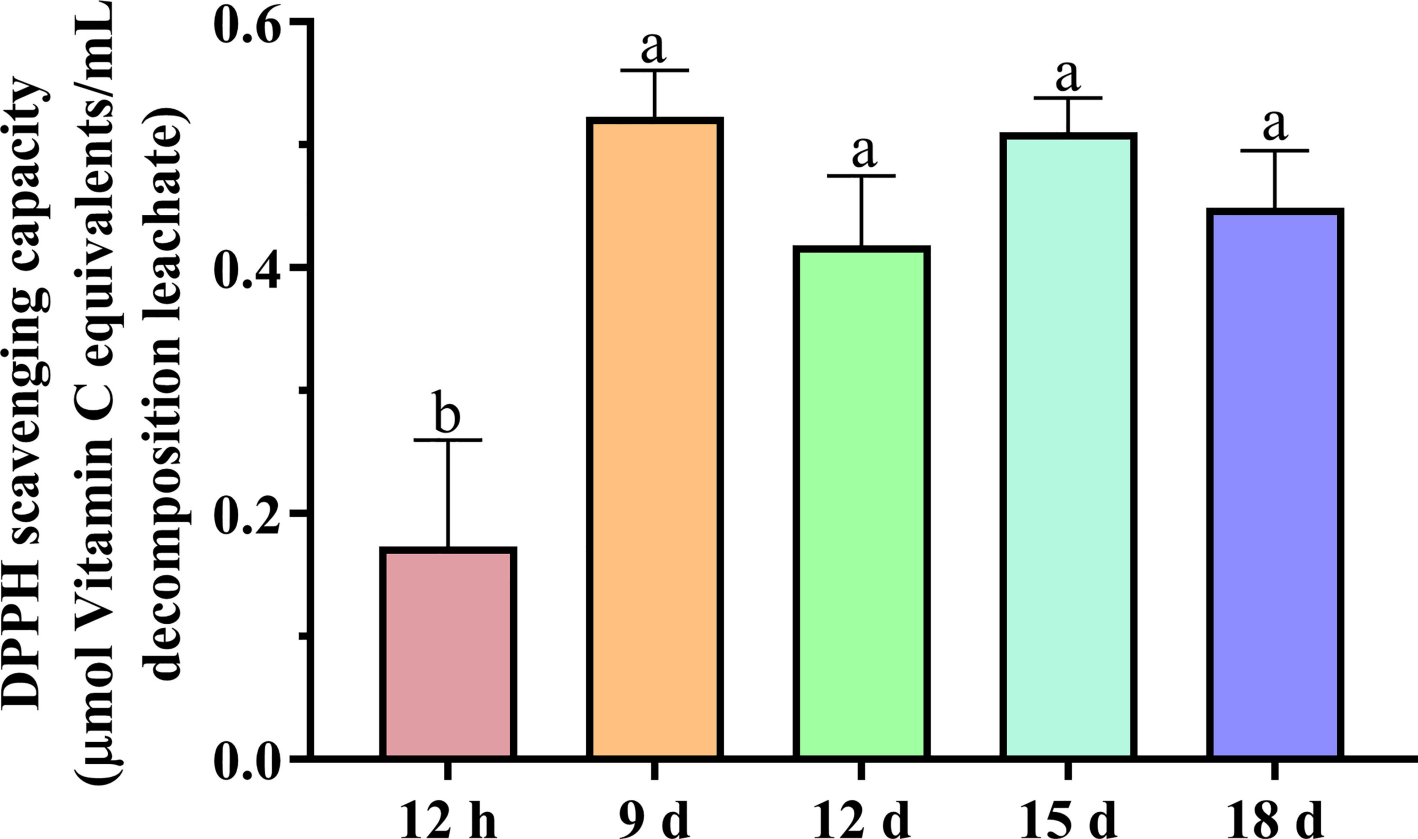
DPPH scavenging capacity of the decomposition leachates at different decay times. Different letters denoted significantly different (*P* < 0.05) among different decomposition leachates of decay durations. Bars represent three repetitions ± SD.

The 12-h decomposition leachate was found to have the lowest DPPH-free radical scavenging ability (0.17 μmol of vitamin C/ml decomposition leachate) (**Figure 6**). DPPH radical scavenging capacity of the decomposition leachates increases with increasing decomposition time. The DPPH radical scavenging capacity in 9-, 12-, 15-, and 18-day decomposition leachates increased to 0.52, 0.42, 0.51, and 0.45 μmol of vitamin C/ml decomposition leachate, respectively.

The presence of alkaloids, flavonoids, glycosides, phenols, steroids, saponins, tannins, anthocyanins, and coumarins in most plant extracts exhibits significant antioxidant and antimicrobial potential (Iram et al., 2019). The type and quantity of extracted compounds and their antioxidant properties vary depending on the plant, plant species, plant organ, soil fertility level, as well as the solvent and extraction method (Toppo et al., 2019; Shah et al., 2019; Sroka et al., 2019; Sarker et al., 2022). In the fermentation tests on defatted barley, the fermentation time led to an increase in DPPH and ABTS^+^ radical scavenging activity, and ferric reducing antioxidant power (Wang et al., 2020), which is consistent with the present study. Similar results were obtained in the fermentation trials with caper berries (*Capparis spinosa* L.), where the glucocapparin, the main component present in the non-fermented fruit, was degraded during the fermentation process, whereas the total phenolic content and antioxidant capacity were increased (Jiménez-López et al., 2018). This is similar to the results of this test where the total phenolic content decreased and the antioxidant activity increased.

### 3.5 Changes in acid fractions and their content in different decay duration decomposition leachates

Liquid chromatography (LC) analysis revealed that the identified acid fractions showed variation at different decomposition times (**Figures S2**, **S3**). The highest total acid content was found in the 12-day decomposition leachate at 4147.7 µg ml^−1^, followed by 12 h (3,162.5 µg ml^−1^) and 15 days (3,141.9 µg ml^−1^), and the lowest at 1,868.1 µg ml^−1^ for 9 days. The highest oxalic acid concentration of 341.2 µg ml^−1^ was recorded in the 12-h treatment and fluctuated with increasing decomposition time, with the lowest concentration of 43.2 µg ml^−1^ at 9 days. The highest tartaric acid content of 429.1 µg ml^−1^ was found in the 12-day decomposition leachates, followed by the 12-h decomposition leachates (279.0 µg ml^−1^), whereas no tartaric acid was detected in the 18-day decomposition. Within 15 days, the acetic acid concentrations increased with increased decomposition time, from 740.3 to 2,308.1 µg ml^−1^, and decreased to 2,126.4 µg ml^−1^ on 18 days. The highest citric acid content of 1,436.4 µg ml^−1^ was recorded in the 12-h decomposition leachate, followed by 12-day decomposition leachate, with a concentration of 420.1 µg ml^−1^. The highest level of succinic acid was found in the 12-h decomposition leachate, reaching 348.1 µg ml^−1^, followed by 12 days (246.1 µg ml^−1^), and the lowest concentration was found in 18 days, at 64.4 µg ml^−1^. No fumaric acid was detected in the 12-h decomposition solution, and the highest fumaric acid content was recorded in the 15-day decomposition solution, reaching 1.1 µg ml^−1^. Within 12 days, the propionic acid content increased with increasing decay time, with the highest propionic acid content (1335.1 µg ml^−1^) in the 12-day decomposition leachate. Beyond 12 days, the propionic acid content of the decomposition leachates decreased, with 53.9 µg ml^−1^ of propionic acid at 18 days. The tannic acid content was highest in the 12-day decomposition leachates, reaching 3.0 µg ml^−1^, followed by the 12-h, 9-day, and 15-day decomposition leachates, with concentrations of 2.1, 1.8, and 0.8 µg ml^−1^, respectively, and no tannic acid was detected in the 18-day decomposition leachate (**Table 3**). From the data on acid content and type, we can tentatively conclude that the decrease in soil pH caused by the return of milk vetch to the field in the previous study is related to the acid produced by decomposition. The incomplete agreement may be due to the unmeasured acid fractions and acidic substances such as 4-ethylphenol, as well as the magnitude of acidity between the acid fractions.

**Table 3:** Acid fractions and concentration in different decomposition leachates.

Compound	Content (µg ml^−1^)				
	12 h	9 days	12 days	15 days	18 days
Oxalic acid	341.2 ± 17.2 a	43.2 ± 2.2 c	85.1 ± 4.3 b	45.1 ± 2.3 c	83.1 ± 4.2 b
Tartaric acid	279.0 ± 78.6 b	53.4 ± 14.9 c	429.1 ± 120.9 a	27.6 ± 7.7 c	n.d. c
Acetic acid	751.4 ± 9.9 d	740.3 ± 1.5 d	1628.0 ± 16.2 c	2308.1 ± 6.6 a	2,126.4 ± 47.5 b
Citric acid	1436.4 ± 12.1 a	254.1 ± 2.1 c	420.1 ± 3.8 b	182.1 ± 1.6 e	219.7 ± 1.9 d
Succinic acid	348.1 ± 6.5 a	119.0 ± 2.3 c	246.1 ± 4.6 b	67.0 ± 1.3 d	64.4 ± 1.2 d
Fumaric acid	4.3 ± 0.04 a	0.1 ± 0.003 d	1.1 ± 0.006 b	0.3 ± 0.02 d	0.7 ± 0.02 c
Propionic acid	n.d. e	656.1 ± 25.9 b	1,335.1 ± 53.3 a	510.9 ± 20.2 c	53.9 ± 2.1 d
Tannic acid	2.1 ± 0.01 b	1.8 ± 0.03 c	3.0 ± 0.08 a	0.8 ± 0.00 d	n.d. e
Total (∑)	3162.5	1868.1	4147.7	3141.9	2,548.2

Data represent mean values of three replicates ± SD. Different letters denoted significantly different (P<0.05) among different decomposition leachates of decay durations.

“n.d.” indicates that the test and result are below the method detection limit. The detection limits (DL) of tartaric acid, fumaric acid, and tannic acid were 1.7, 0.8, and 0.8 µg ml^−1^ respectively. Statistical calculations were performed using one-half of the DL concentration.

The acid fractions measured in this experiment are consistent with Gu et al. (2021); the proportion of citric, malic, and oxalic acids in the total organic acids of six green manure crop decompositions was closely related to the activation level of Al-P; the proportion of tartaric acid was related to the activation level of Fe-P, whereas milk vetch, which contains higher amounts of oxalic, citric, and succinic acids, had stronger activation of Al-P (Gu et al., 2021). Similar studies have shown that the return of leguminous green manures such as milk vetch and hairy vetch to the field can reduce soil pH at the initial stage and rebounded at a later stage; the remaining three types of green manure, however, do not lead to such a result (Su et al., 2022b), which is consistence with our study. Within reasonable limits, a lower pH value facilitates nutrient uptake by plants. Woody composts are more suitable for use in growing media due to their lower pH, EC, and inorganic C content comparison with regular green manure composts (Vandecasteele et al., 2022). This study was carried out under laboratory conditions and further research is needed to determine whether it has an effect on soil pH in the field.

Green manure decomposition is a simultaneous process of decomposition and synthesis, as shown by the changes in the content of the four goosegrass-resistant active substances and the acid fractions. The single components of the four goosegrass-resistant active substances have less effect on the germination and growth of goosegrass compared with the mixture treatment and do not fully reproduce the effect of the decomposition leachate treatment. In experiments exploring possible synergies between volatile organic compounds (VOCs) and water-soluble compounds released into the soil matrix by *Cytisus scoparius*, the presence of the aqueous extract significantly increased the phytotoxicity of volatile on weed root growth compared with the volatiles emitted alone. In addition, soil enhanced the synergistic interactions among VOCs and water-soluble compounds (Pardo Muras et al., 2022). In this study, the presence of the high concentration of phenolic and antioxidant activity in the decomposition leachates and therefore whether the presence of these compounds may reduce the goosegrass suppression effect of the four goosegrass-resistant active substances need to be further investigated, as well as whether the organic acids present in the decomposition leachates may also affect the effect of goosegrass-resistant and their effect on the validity of nutrients in the soil and therefore crop growth.

## 4 Conclusion

In this study, we elucidated the reasons why the incorporation of milk vetch can inhibit goosegrass growth from the perspective of the changes of the chemical compound in different decomposition leachates. The 15-day milk vetch decomposition leachate had the strongest suppression capacity of goosegrass, which related to the increased concentrations of the four qualitative goosegrass-resistant potential substances, particularly N-acrylimorpholine and 2-hydroxyethyl acrylate, with N-acrylimorpholine at the highest concentration of 247.2 µg ml^−1^ at 18 days and 2-hydroxyethyl acrylate at the highest concentration of 127.1 µg ml^−1^ at 15 days. Phenolic and flavonoid compounds in the decomposing leachates were analyzed as possible crop growth promoters, their concentrations being highest at the initial stage (12 h) at 532.3 and 72.1 mg L^−1^, respectively, and decreased with decay time. Finally, the content of organic acid fractions in the decomposition leachates and their variation preliminarily explained the reasons for the changes in pH of the decomposition leachates at different decomposition days, which can be combined with specific agronomic measures to suppress the decrease of soil pH during decomposition and provide a reference for the better use of milk vetch as high-quality green manure.

## Data availability statement

The raw data supporting the conclusions of this article will be made available by the authors, without undue reservation.

## Author contributions

SL and YM: conceptualization. SL and WW: experimental method and design, software, original draft, and writing. YX, YZ, ZC, and XD: data curation and revision of the manuscript. YM and ZM: funding acquisition, supervision, and validation. All authors have read and agreed to the published version of this manuscript.

## Funding

This project was supported by Natural Science Foundation of Guangdong Province (2018A0303130194) and Bayer Crop Science supporting project (2021 Grants4AG).

## Conflict of interest

The authors declare that the research was conducted in the absence of any commercial or financial relationships that could be construed as a potential conflict of interest.

## Publisher’s note

All claims expressed in this article are solely those of the authors and do not necessarily represent those of their affiliated organizations, or those of the publisher, the editors and the reviewers. Any product that may be evaluated in this article, or claim that may be made by its manufacturer, is not guaranteed or endorsed by the publisher.
