# Supplementary material for: Weed suppression and antioxidant activity of Astragalus sinicus L. decomposition leachates
Source: Front Plant Sci. 2022 Nov 16;13:1013443. doi: 10.3389/fpls.2022.1013443 (PMC9709434; doi:10.3389/fpls.2022.1013443)
Supplement: Supplementary file 1 [file DataSheet_1.docx]

**Figure S1.** LC weed suppression active substances profile of different decay duration of milk vetch decomposition leachates; STD/STDs, standard/standards.

**

**

**Figure S2.** LC acid fractions profile of different decomposition times milk vetch decomposition leachates

**

**

**Figure S3.** LC of acid standards; STDs, standards.


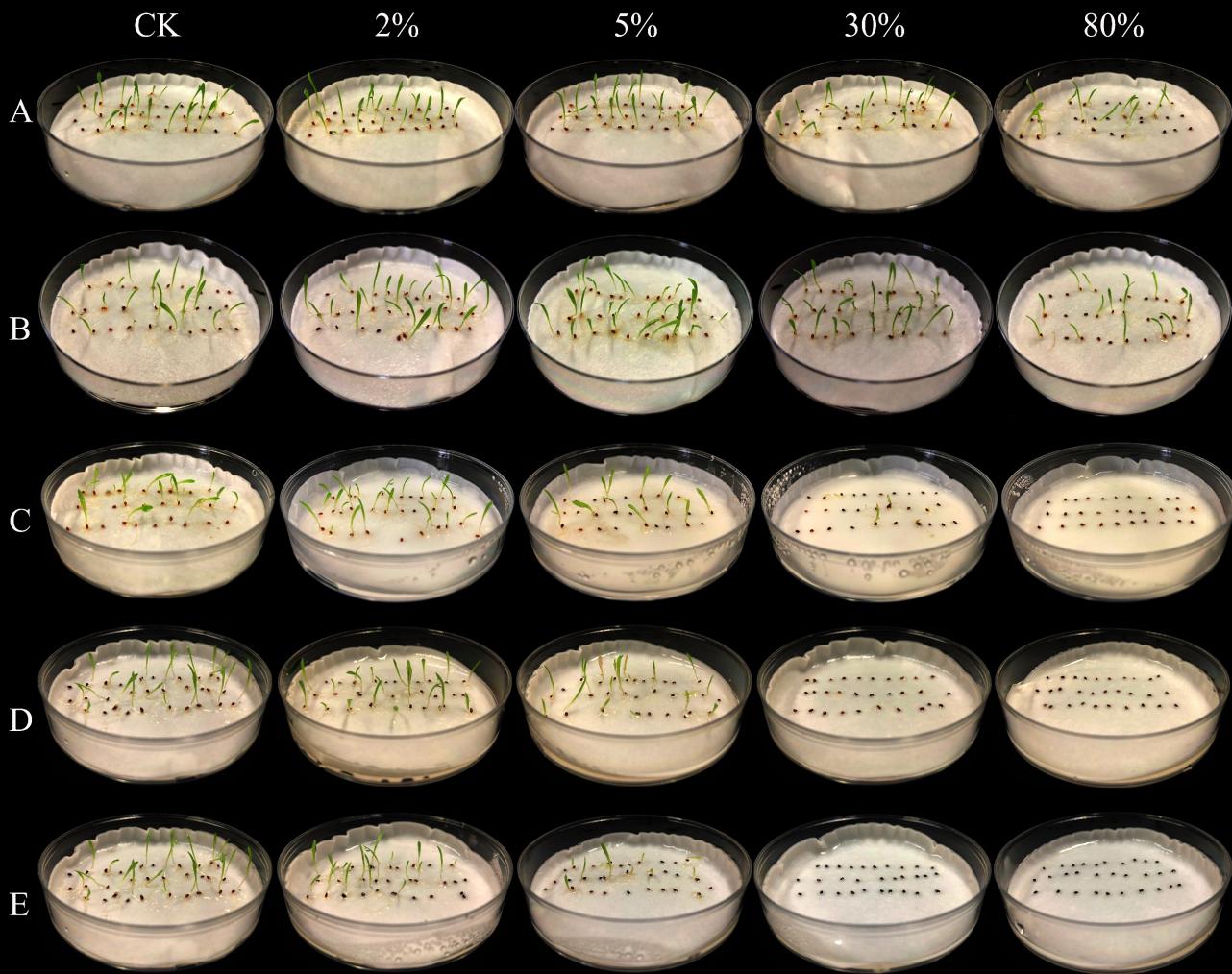


**Picture S1.** Effect of different identified substances on germination and growth of goosegrass

A: 4-ethylphenol; B: Allyl isothiocyanate; C: N-acrylimorpholine; D: 2-hydroxyethyl acrylate; E: Mixture of A-D.
